# Effect of delayed hospitalization on patients with non-ST-segment elevation myocardial infarction and complex lesions undergoing successful new-generation drug-eluting stents implantation

**DOI:** 10.1038/s41598-023-43385-3

**Published:** 2023-09-26

**Authors:** Yong Hoon Kim, Ae-Young Her, Seung-Woon Rha, Cheol Ung Choi, Byoung Geol Choi, Ji Bak Kim, Soohyung Park, Dong Oh Kang, Ji Young Park, Woong Gil Choi, Sang-Ho Park, Myung Ho Jeong

**Affiliations:** 1 Division of Cardiology, Department of Internal Medicine, Kangwon National University College of Medicine, Kangwon National University School of Medicine, 156 Baengnyeong Road, Chuncheon City, Gangwon Province 24289 Republic of Korea; 2 Cardiovascular Center, Korea University Guro Hospital, 148, Gurodong-Ro, Guro-Gu, Seoul, 08308 Republic of Korea; 3 Cardiovascular Research Institute, Korea University College of Medicine, Seoul, Republic of Korea; 4 Division of Cardiology, Department of Internal Medicine, Nowon Eulji Medical Center, Eulji University, Cardiovascular Center, Seoul, Republic of Korea; 5 Division of Cardiology, Department of Internal Medicine, College of Medicine, Chungbuk National University Hospital, Chungbuk National University, Cheongju, Republic of Korea; 6 Cardiology Department, Soonchunhyang University Cheonan Hospital, Cheonan, Republic of Korea; 7 Department of Cardiology, Cardiovascular Center, Chonnam National University Hospital, Gwangju, Republic of Korea

**Keywords:** Cardiology, Diseases

## Abstract

In the absence of available data, we evaluated the effects of delayed hospitalization (symptom-to-door time [SDT] ≥ 24 h) on major clinical outcomes after new-generation drug-eluting stent implantation in patients with non-ST-segment elevation myocardial infarction (NSTEMI) and complex lesions. In total, 4373 patients with NSTEMI were divided into complex (n = 2106) and non-complex (n = 2267) groups. The primary outcome was the 3-year rate of major adverse cardiac events (MACE), defined as all-cause death, recurrent MI, and any repeat revascularization. Secondary outcomes included the individual MACE components. In the complex group, all-cause death (adjusted hazard ratio [aHR], 1.752; p = 0.004) and cardiac death (aHR, 1.966; p = 0.010) rates were significantly higher for patients with SDT ≥ 24 h than for those with SDT < 24 h. In the non-complex group, all patients showed similar clinical outcomes. Patients with SDT < 24 h (aHR, 1.323; p = 0.031) and those with SDT ≥ 24 h (aHR, 1.606; p = 0.027) showed significantly higher rates of any repeat revascularization and all-cause death, respectively, in the complex group than in the non-complex group. Thus, in the complex group, delayed hospitalization was associated with higher 3-year mortalities.

For patients with ST-segment elevation myocardial infarction (STEMI), rapid restoration of blood flow in the infarct-related artery (IRA) is critical to salvage the ischemic myocardium and improve long-term mortality^1,2^. Therefore, primary percutaneous coronary intervention (PPCI) is recommended for all STEMI patients presenting within 12 h of symptom onset^1,2^. However, published data concerning the effects of PCI on long-term clinical outcomes in STEMI patients who present > 12 h after symptom onset (latecomers) is inconsistent^3–5^. Previous reports have emphasized the importance of shortening the door-to-balloon time (DBT, < 60 min) to improve survival in patients with STEMI^6,7^. A recent report^8^ showed that DBT did not affect mortality in 4839 PPCI‐treated patients. Because the DBT interval occupies a later period in the flat slope of the time-myonecrosis curve, reperfusion has a small impact on myocardial salvage during this period; the total ischemic time becomes more important^8^. Considering these observations^8^, the symptom-to-door time [SDT] can be more important than DBT^9^. With regard to non-STEMI (NSTEMI), a previous study^9^ including patients with NSTEMI found that patients with delayed hospitalization (SDT ≥ 24 h) exhibited a higher 3-year mortality rate than did those without delayed hospitalization (SDT < 24 h) (17.0% vs. 10.5%, p < 0.001). However, approximately 15% of the study population did not receive PCI or had underwent unsuccessful PCI, and patients who received bare-metal stents or first-generation drug-eluting stents (DES) were included^9^. Moreover, some important laboratory results of patients with acute myocardial infarction (AMI), such as cardiac biomarkers, the lipid profile, and serum creatinine levels, were not included in the baseline characteristics of the study population. Because of these limitations, this study^9^ could not accurately reflect current real-world practice. To the best of our knowledge, there are no large-scale studies comparing long-term clinical outcomes according to the presence or absence of delayed hospitalization in patients with NSTEMI and complex lesions. Compared to non-complex lesions, PCI for complex lesions frequently requires extended procedural durations and specialized interventional skills and techniques, and is often associated with the risk of hemodynamic instability^10^. Therefore, considering the association between increased mortality and delayed hospitalization in patients with NSTEMI^9^, we investigated the impact of delayed hospitalization on clinical outcomes in complex and non-complex groups, as well as the total study population in order to provide a more precise understanding of the significance of delayed hospitalization in patients with NSTEMI. We also attempted to identify independent predictors of poorer clinical outcomes in NSTEMI patients with complex lesions, with the aim of effectively addressing and managing these independent predictors to improve the clinical outcomes of these patients. To reflect current real-world practice, we limited the study population to patients with NSTEMI who received successful new-generation DES implantation. Finally, according to the presence or absence of complex lesions, we compared clinical outcomes between NSTEMI patients with SDT < 24 h and those with SDT ≥ 24 h who received successful new-generation DES implantation.

## Results

### Baseline characteristics

**Figure 1:**
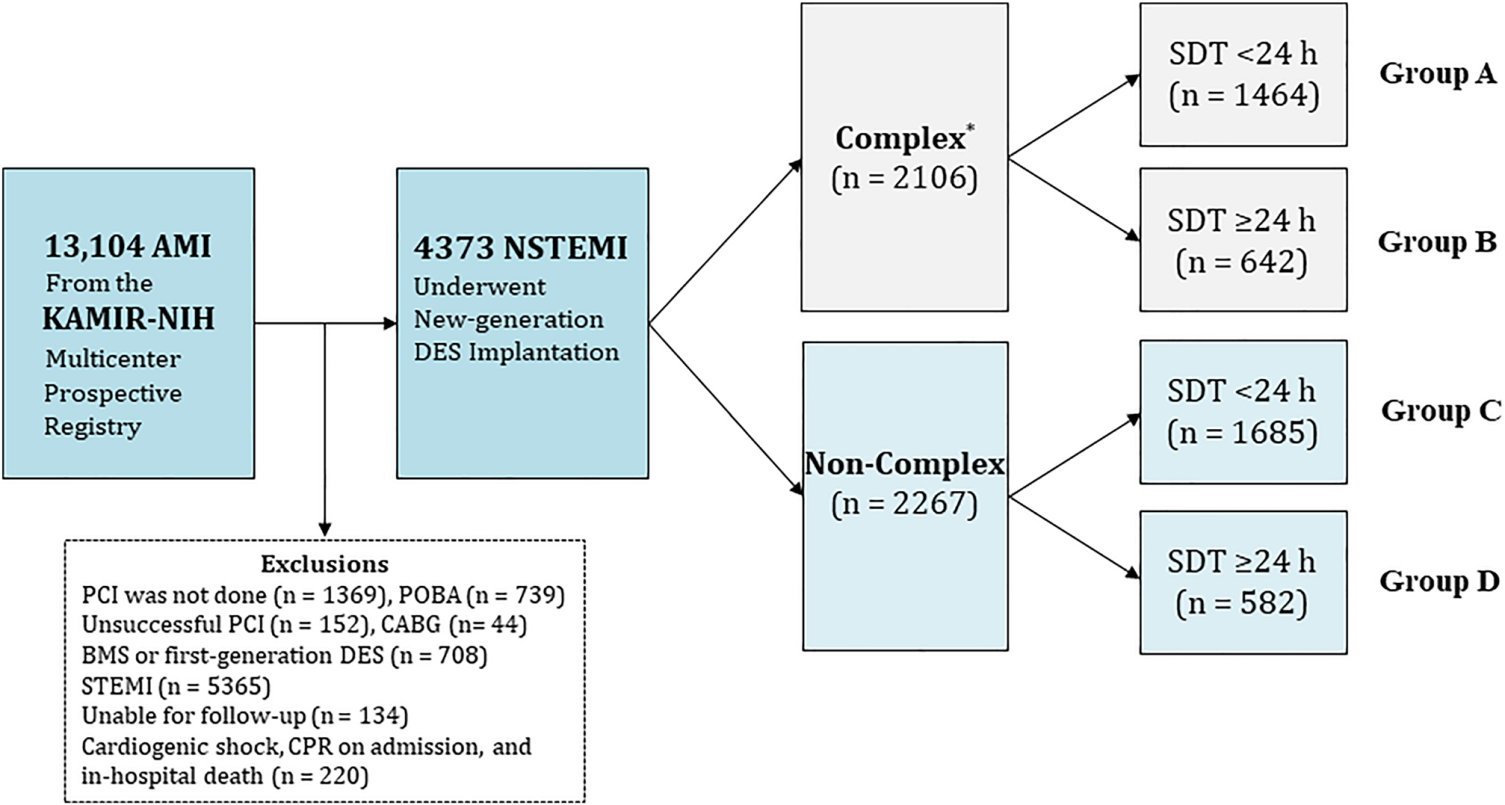
Flowchart. *AMI* acute myocardial infarction, *KAMIR-NIH* Korea Acute Myocardial Infarction Registry-National Institute of Health, *PCI* percutaneous coronary intervention, *POBA* plain old balloon angioplasty, *CABG* coronary artery bypass graft, *BMS* bare-metal stent, *DES* drug-eluting stent, *STEMI* ST-segment-elevation myocardial infarction, *NSTEMI* non-STEMI, *CPR* cardiopulmonary resuscitation, *SDT* symptom-to-door time. Complex lesions were defined as PCI for unprotected left main coronary disease, multivessel PCI, multiple stents implantation (≥ 3 stents per patient), or those with the total length of deployed stent being over 38 mm.

Figure 1 shows the study flowchart. Table 1 shows the baseline characteristics of the SDT < 24 h and SDT ≥ 24 h groups according to the presence or absence of complex lesions. In both the NSTEMI and complex group (complex group) and NSTEMI and non-complex group (non-complex group), the number of male patients, current smokers, and patients who used emergency medical services (EMS) to arrive at the hospital; the mean systolic blood pressure and diastolic blood pressure values; and peak creatine kinase myocardial band (CK-MB), troponin-I, and blood glucose levels were higher for patients with SDT < 24 h than for patients with SDT ≥ 24 h. In contrast, the mean age; the mean Global Registry of Acute Coronary Events (GRACE) risk score; the number of patients with atypical chest pain and dyspnea, Killip class II/III, hypertension, and diabetes mellitus; and number of people who visited hospitals incapable of performing PCI were higher in the SDT ≥ 24 h group than in the SDT < 24 h group. Table S1 in the Supplementary Appendix shows the baseline characteristics in the complex and non-complex groups according to the presence or absence of delayed hospitalization. For both patients with SDT < 24 h and those with SDT ≥ 24 h, the mean age; the number of patients with Killip class II/III, hypertension, diabetes mellitus, and the American College of Cardiology/American Heart Association (ACC/AHA) type B2/C lesions; and mean blood glucose levels were higher in the complex group than in the non-complex group. However, the mean left ventricular ejection fraction (LVEF) was higher in the non-complex group than in the complex group. Table S2 in the Supplementary Appendix compares the baseline characteristics between the SDT < 24 h and SDT ≥ 24 h groups in the total study population and propensity score (PS)-matched population.

**Table 1:** Baseline characteristics between the SDT < 24 h and SDT ≥ 24 h groups according to the presence or absence of complex lesions.

Variables	Complex (n = 2106)			Non-complex (n = 2267)		
	SDT < 24 h (n = 1464, group A)	SDT ≥ 24 h (n = 642, group B)	p value	SDT < 24 h (n = 1685, group C)	SDT ≥ 24 h (n = 582, group D)	p value
Male, n (%)	1082 (73.9)	422 (65.7)	< 0.001	1295 (76.9)	399 (68.6)	< 0.001
Age, years	64.6 ± 11.6	67.2 ± 11.5	< 0.001	61.8 ± 12.1	65.3 ± 12.2	< 0.001
LVEF, %	54.3 ± 10.2	53.0 ± 11.2	0.012	55.6 ± 9.5	54.6 ± 11.0	0.050
BMI, kg/m^2^	24.2 ± 3.3	24.0 ± 3.7	0.348	24.2 ± 3.3	24.0 ± 3.2	0.350
SBP, mmHg	138.2 ± 26.2	134.6 ± 23.5	0.002	137.8 ± 25.6	134.8 ± 23.1	0.009
DBP, mmHg	81.9 ± 14.9	80.1 ± 14.0	0.007	83.2 ± 15.5	81.7 ± 13.4	0.022
SDT, hours	4.0 (1.8–8.6)	72.0 (35.6–161.4)	< 0.001	3.8 (1.6–8.2)	71.4 (33.6–120.0)	< 0.001
DBT, hours	14.9 (4.1–26.7)	16.4 (4.0–24.9)	0.162	12.2 (3.9–23.9)	16.9 (4.0–27.1)	0.001
SBT, hours	20.9 (7.2–83.0)	90.2 (51.2–186.2)	< 0.001	18.1 (9.3–30.9)	88.9 (51.6–159.5)	< 0.001
Atypical chest pain, n (%)	185 (12.6)	149 (23.2)	< 0.001	187 (11.1)	117 (20.1)	< 0.001
Dyspnea, n (%)	341 (23.3)	199 (31.0)	< 0.001	327 (19.4)	151 (25.9)	0.001
EKG on admission						
Q-wave, n (%)	103 (7.0)	69 (10.7)	0.006	112 (6.6)	58 (10.0)	0.011
ST-segment depression, n (%)	374 (25.5)	128 (19.9)	0.005	350 (20.8)	98 (16.8)	0.040
T-wave inversion, n (%)	336 (23.0)	175 (27.3)	0.036	349 (20.7)	166 (28.5)	< 0.001
Atrial fibrillation, n (%)	53 (3.6)	28 (4.4)	0.460	60 (3.6)	18 (3.1)	0.693
Killip class 1I/III, n (%)	240 (16.4)	137 (21.3)	0.008	180 (10.7)	84 (14.4)	0.017
First medical contact						
EMS, n (%)	176 (12.0)	27 (4.2)	< 0.001	203 (12.0)	15 (2.6)	< 0.001
Non-PCI center, n (%)	752 (51.4)	380 (59.2)	0.001	838 (49.7)	338 (58.1)	0.001
PCI center, n (%)	536 (36.6)	235 (36.6)	0.997	644 (38.2)	229 (39.3)	0.657
Hypertension, n (%)	798 (54.5)	388 (60.4)	0.013	821 (48.7)	304 (52.2)	0.149
Diabetes mellitus, n (%)	472 (32.2)	251 (39.1)	0.003	413 (24.5)	168 (28.9)	0.042
Dyslipidemia, n (%)	179 (12.2)	75 (11.7)	0.771	208 (12.3)	67 (11.5)	0.659
Previous MI, n (%)	98 (6.7)	46 (7.2)	0.708	115 (6.8)	40 (6.9)	0.968
Previous PCI, n (%)	146 (10.0)	60 (9.3)	0.691	178 (10.6)	51 (8.8)	0.232
Previous CABG, n (%)	10 (0.7)	5 (0.8)	0.783	11 (0.7)	6 (1.0)	0.403
Previous HF, n (%)	24 (1.6)	11 (1.7)	0.855	18 (1.1)	8 (1.4)	0.507
Previous stroke, n (%)	80 (5.5)	52 (8.1)	0.025	80 (4.7)	34 (5.8)	0.322
Current smokers, n (%)	518 (35.4)	170 (26.5)	< 0.001	694 (41.2)	204 (35.1)	0.009
Peak CK-MB, mg/dL	24.2 (7.3–82.4)	11.4 (4.8–33.5)	0.016	27.1 (6.7–95.3)	11.9 (4.1–41.2)	< 0.001
Peak troponin-I, ng/mL	9.1 (2.0–23.0)	4.9 (1.5–13.7)	< 0.001	11.0 (2.0–23.0)	4.08 (1.0–13.7)	< 0.001
Blood glucose, mg/dL	165.5 ± 82.0	153.6 ± 58.8	0.001	152.5 ± 67.3	145.2 ± 74.1	0.036
Serum creatinine (mg/L)	1.13 ± 1.20	1.21 ± 1.31	0.190	1.07 ± 1.30	1.11 ± 1.35	0.572
Total cholesterol, mg/dL	179.0 ± 43.4	176.3 ± 44.2	0.186	182.8 ± 44.2	177.3 ± 44.6	0.010
Triglyceride, mg/L	130.2 ± 103.8	128.8 ± 107.6	0.795	139.0 ± 129.2	128.5 ± 79.8	0.022
HDL cholesterol, mg/L	42.5 ± 11.4	41.5 ± 11.3	0.076	43.8 ± 11.1	42.5 ± 11.6	0.021
LDL cholesterol, mg/L	113.2 ± 38.6	111.5 ± 36.5	0.308	115.7 ± 36.0	111.8 ± 35.9	0.025
GRACE risk score	131.1 ± 38.3	135.4 ± 35.2	0.014	120.6 ± 35.6	127.4 ± 32.5	< 0.001
Discharge medications, n (%)						
Aspirin, n (%)	1455 (99.4)	636 (99.1)	0.410	1676 (99.5)	574 (98.6)	0.052
Clopidogrel, n (%)	1020 (69.7)	469 (73.1)	0.119	1210 (71.8)	441 (75.8)	0.066
Ticagrelor, n (%)	301 (20.6)	116 (18.1)	0.192	323 (19.2)	88 (15.1)	0.029
Prasugrel, n (%)	143 (9.8)	57 (8.9)	0.572	152 (9.0)	53 (9.1)	0.933
BBs, n (%)	1274 (87.0)	552 (86.0)	0.531	1439 (85.4)	486 (83.5)	0.283
ACEI or ARBs, n (%)	1218 (83.2)	527 (82.1)	0.531	1416 (84.0)	478 (82.1)	0.300
Statin, n (%)	1401 (95.7)	609 (94.9)	0.427	1613 (95.7)	555 (95.4)	0.724
Anticoagulant, n (%)	20 (1.4)	21 (3.3)	0.006	30 (1.8)	15 (2.6)	0.231
Infarct-related artery						
Left main, n (%)	80 (5.5)	45 (7.0)	0.192	–	–	–
LAD, n (%)	598 (40.8)	259 (40.3)	0.847	748 (44.4)	257 (44.2)	0.961
LCx, n (%)	341 (23.3)	120 (18.7)	0.019	502 (29.8)	161 (27.7)	0.342
RCA, n (%)	445 (30.4)	218 (34.0)	0.114	435 (25.8)	164 (28.2)	0.276
Treated vessel						
Left main, n (%)	122 (8.3)	73 (11.4)	0.033	–	–	–
LAD, n (%)	1038 (70.9)	466 (72.6)	0.463	748 (44.4)	257 (44.2)	0.961
LCx, n (%)	724 (49.5)	304 (47.4)	0.394	502 (29.8)	161 (27.7)	0.342
RCA, n (%)	716 (48.9)	329 (51.2)	0.344	435 (25.8)	164 (28.2)	0.276
ACC/AHA type B2/C lesions, n (%)	1293 (88.3)	575 (89.6)	0.455	1348 (80.0)	453 (77.8)	0.284
Pre-PCI TIMI flow grade 0/1, n (%)	563 (38.5)	256 (39.9)	0.560	646 (38.3)	207 (35.6)	0.254
GP IIb/IIIa inhibitor, n (%)	140 (9.6)	61 (9.5)	0.985	127 (7.5)	44 (7.6)	0.986
Transradial approach, n (%)	717 (49.0)	345 (53.7)	0.047	916 (54.4)	350 (60.1)	0.016
IVUS/OCT, n (%)	441 (28.1)	177 (27.6)	0.833	385 (22.8)	138 (23.7)	0.690
FFR, n (%)	41 (2.8)	19 (3.0)	0.887	33 (2.0)	7 (1.2)	0.276
Drug-eluting stents*						
ZES, n (%)	329 (22.5)	123 (19.2)	0.095	426 (25.3)	126 (21.6)	0.083
EES, n (%)	871 (59.5)	399 (62.1)	0.266	773 (45.9)	254 (43.6)	0.359
BES, n (%)	203 (13.9)	98 (15.3)	0.762	427 (25.3)	181 (31.1)	0.008
Others, n (%)	61 (4.2)	22 (3.4)	0.467	59 (3.5)	21 (3.6)	0.904
Stent diameter (mm)	3.06 ± 0.40	3.04 ± 0.40	0.200	3.10 ± 0.44	3.10 ± 0.44	0.761
Stent length (mm)	36.8 ± 15.9	37.3 ± 16.5	0.567	22.9 ± 6.09	21.8 ± 5.93	< 0.001
Number of stents	1.40 ± 0.57	1.38 ± 0.56	0.642	1.03 ± 0.19	1.02 ± 0.18	0.411

Values are means ± standard deviation or median (interquartile range) or numbers and percentages. The p values for continuous data were obtained from the unpaired t-test. The p values for categorical data from chi-square or Fisher’s exact test. *SDT* symptom-to-door time, *LVEF* left ventricular ejection fraction, *BMI* body mass index, *SBP* systolic blood pressure, *DBP,* diastolic blood pressure, *PCI* percutaneous coronary intervention, *MI* myocardial infarction, *CABG* coronary artery bypass graft, *HF* heart failure, *CK-MB* creatine kinase myocardial band, *Hs-CRP* high sensitivity C-reactive protein, *HDL* high-density lipoprotein, *LDL* low-density lipoprotein, *GRACE* Global Registry of Acute Coronary Events, *BBs* ß-blockers, *ACEIs* angiotensin-converting enzyme inhibitors, *ARBs* angiotensin receptor blockers, LAD left anterior descending artery, *LCx* left circumflex artery, *RCA* right coronary artery, *ACC/AHA* American College of Cardiology/American Heart Association, *TIMI* thrombolysis in myocardial infarction, *GP* glycoprotein, *IVUS* intravascular ultrasound, *OCT* optical coherence tomography, *FFR* fractional flow reserve, *ZES* zotarolimus-eluting stent, *EES* everolimus-eluting stent, *BES* biolimus-eluting stent.

*Drug-eluting stents were composed of ZES (Resolute integrity stent; Medtronic, Inc., Minneapolis, MN), EES (Xience Prime stent, Abbott Vascular, Santa Clara, CA; or Promus Element stent, Boston Scientific, Natick, MA), and BES (BioMatrix Flex stent, Biosensors International, Morges, Switzerland; or Nobori stent, Terumo Corporation, Tokyo, Japan).

### Clinical outcomes

**Figure 2:**
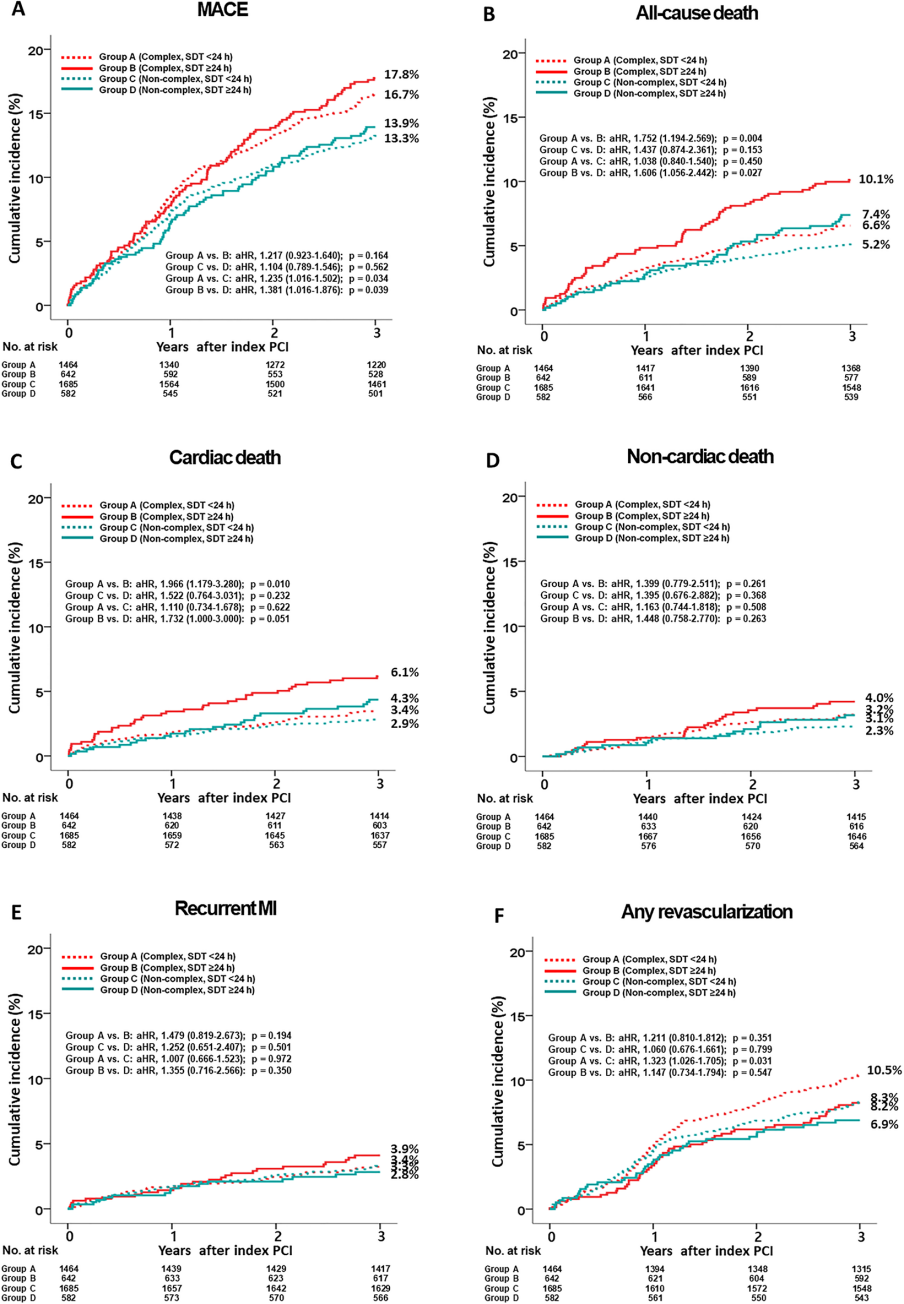
Kaplan–Meier curved analysis for MACE (**A**), all-cause death (**B**), cardiac death (**C**), non-cardiac death (**D**), recurrent MI (**E**), and any repeat revascularization (**F**). *MACE* major adverse cardiac events, *SDT* symptom-to-door time, *aHR* adjusted hazard ratio, *PCI* percutaneous coronary intervention, *MI* myocardial infarction.

**Figure 3:**
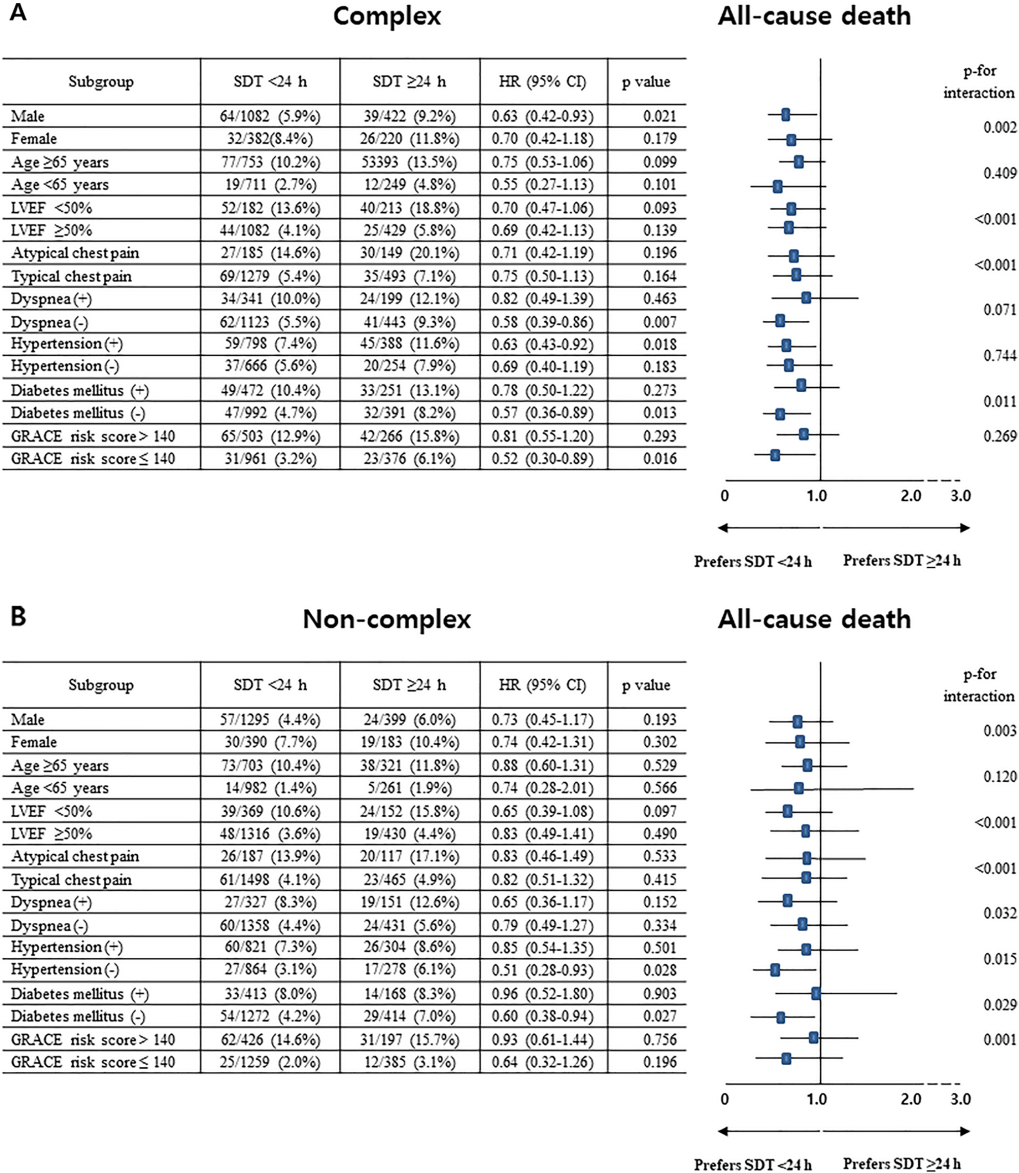
Subgroup analysis for all-cause death in the complex (**A**) and non-complex (**B**) groups. *SDT* symptom-to-door time; *HR* hazard ratio, *CI* confidence interval, *LVEF* left ventricular ejection fraction, *GRACE* Global Registry of Acute Coronary Events.

The 3-year major clinical outcomes are summarized in Tables 2, 3, and Fig. 2. In the complex group, after multivariable-adjusted analyses, the major adverse cardiac events (MACE) rate (adjusted hazard ratio [aHR], 1.217; 95% confidence interval [CI], 0.923–1.640; p = 0.164; Fig. 2A) was similar in the SDT < 24 h and SDT ≥ 24 h groups. However, all-cause death (aHR, 1.752; 95% CI, 1.194–2.569; p = 0.004; Fig. 2B) and cardiac death (CD) (aHR, 1.966; 95% CI, 1.179–3.280; p = 0.010; Fig. 2C) rates were significantly higher for patients with SDT ≥ 24 h than for those with SDT < 24 h. The non-CD (NCD, Fig. 2D), recurrent MI (Fig. 2E), and repeat revascularization (Fig. 2F) rates were not significantly different patients with SDT < 24 h and those with SDT ≥ 24 h (Table 2). In the non-complex group, the primary and secondary outcomes were not significantly different patients with SDT < 24 h and those with SDT ≥ 24 h. In the total study population, the all-cause death (aHR, 1.512; 95% CI 1.125–2.033; p = 0.006) and CD (aHR, 1.614; 95% CI 1.100–2.448; p = 0.015) rates were significantly higher for patients with SDT ≥ 24 h than for those with SDT < 24 h (Table 2). These results were confirmed by PS-adjusted analyses (Table 2). For patients with SDT < 24 h group, multivariable-adjusted analyses showed that MACE (aHR, 1.235; p = 0.034) and repeat revascularization (aHR, 1.323; p = 0.031) rates were significantly higher in the complex group than in the non-complex group (Table 3). For patients with SDT ≥ 24 h, the MACE (aHR, 1.381; p = 0.039) and all-cause death (aHR, 1.606; p = 0.027) rates were significantly higher in the complex group than in the non-complex group (Table 3). When the total study population was considered, MACE (aHR, 1.283; p = 0.003), all-cause death (aHR, 1.303; p = 0.033), and repeat revascularization (aHR, 1.281; p = 0.028) rates were significantly higher in the complex group than in the non-complex group. Figure 3A and B show the results of subgroup analysis of all-cause death. Among patients without dyspnea, those with hypertension, and those with a low GRACE risk score (< 140) in the complex group (Fig. 3A), SDT < 24 h was associated with a lower all-cause death rate than was SDT ≥ 24 h. In the non-complex group (Fig. 3B), all subgroups, except for those showing significant p-for-interaction, demonstrated comparable all-cause death rates between the SDT < 24 h and SDT ≥ 24 h groups. Table 4 shows the independent predictors of all-cause death. In both the complex and non-complex groups with NSTEMI, old age (≥ 65 years), a reduced left ventricular ejection fraction (< 50%), atypical chest pain, and a high GRACE risk score were common independent predictors of all-cause death.

**Table 2:** Clinical outcomes of the SDT < 24 h and SDT ≥ 24 h groups in patient with or without complex lesions at 3 years.

Outcomes	Complex, n = 2106								
	SDT < 24 h (n = 1464, group A)	SDT ≥ 24 h (n = 642, group B)	Log-rank	Unadjusted		Multivariable-adjusted*		Propensity score-adjusted	
				HR (95% CI)	p	HR (95% CI)	p	HR (95% CI)	p
MACE	244 (16.7)	114 (17.8)	0.555	0.935 (0.749–1.168)	0.555	1.217 (0.923–1.640)	0.164	1.153 (0.887–1.499)	0.286
All-cause death	96 (6.6)	65 (10.1)	0.004	0.635 (0.463–0.870)	0.005	1.752 (1.194–2.569)	0.004	1.694 (0.171–2.450)	0.005
Cardiac death	50 (3.4)	39 (6.1)	0.005	0.552 (0.363–0.838)	0.005	1.966 (1.179–3.280)	0.010	1.885 (1.150–3.092)	0.012
Non-cardiac death	46 (3.2)	26 (4.0)	0.260	0.759 (0.469–1.228)	0.262	1.399 (0.779–2.511)	0.261	1.357 (0.775–2.375)	0.286
Recurrent MI	47 (3.3)	25 (3.9)	0.387	0.807 (0.497–1.312)	0.387	1.479 (0.819–2.673)	0.194	1.260 (0.715–2..223)	0.424
Any repeat revascularization	149 (10.5)	50 (8.2)	0.108	1.299 (0.943–1.790)	0.109	1.211 (0.810–1.812)	0.351	1.240 (0.850–1.829)	0.265

Outcomes	Non-complex, n = 2267								
	SDT < 24 h (n = 1685, group C)	SDT ≥ 24 h (n = 582, group D)	Log-rank	Unadjusted		Multivariable-adjusted*		Propensity score-adjusted	
				HR (95% CI)	p	HR (95% CI)	p	HR (95% CI)	p
MACE	224 (13.3)	81 (13.9)	0.741	0.958 (0.743–1.235)	0.741	1.104 (0.789–1.546)	0.562	1.082 (0.772–1.480)	0.620
All-cause death	87 (5.2)	43 (7.4)	0.050	0.695 (0.482–1.002)	0.051	1.437 (0.874–2.361)	0.153	1.550 (0.977–2.459)	0.063
Cardiac death	48 (2.9)	25 (4.3)	0.090	0.660 (0.407–1.070)	0.092	1.522 (0.764–3.031)	0.232	1.616 (0.870–3.102)	0.129
Non-cardiac death	39 (2.3)	18 (3.1)	0.297	0.744 (0.426–1.300)	0.299	1.395 (0.676–2.882)	0.368	1.479 (0.740–2.957)	0.269
Recurrent MI	56 (3.4)	16 (2.8)	0.507	1.207 (0.692–2.103)	0.508	1.252 (0.651–2.407)	0.501	1.477 (0.763–2.858)	0.247
Any repeat revascularization	137 (8.3)	39 (6.9)	0.277	1.218 (0.853–1.738)	0.278	1.060 (0.676–1.661)	0.799	1.174 (0.770–1.790)	0.456

Outcomes		Total, n = 4373							
	SDT < 24 h (n = 3149, group A + C)	SDT ≥ 24 h (n = 1224, group B + D)	Log-rank	Unadjusted		Multivariable-adjusted*		Propensity score-adjusted	
				HR (95% CI)	p	HR (95% CI)	p	HR (95% CI)	p
MACE	468 (14.9)	195 (15.9)	0.407	0.932 (0.788–1.101)	0.407	1.136 (0.921–1.401)	0.232	1.099 (0.902–1.338)	0.349
All-cause death	183 (5.8)	108 (8.8)	< 0.001	0.650 (0.512–0.824)	< 0.001	1.512 (1.125–2.033)	0.006	1.546 (1.167–2.048)	0.002
Cardiac death	98 (3.1)	64 (5.3)	0.001	0.588 (0.429–0.805)	0.001	1.614 (1.100–2.448)	0.015	1.669 (1.144–2.435)	0.008
Non-cardiac death	85 (2.7)	44 (3.7)	0.104	0.740 (0.514–1.065)	0.105	1.325 (0.854–2.055)	0.209	1.349 (0.886–2.145)	0.163
Recurrent MI	103 (3.4)	41 (3.5)	0.847	0.965 (0.672–1.386)	0.847	1.122 (0.724–1.738)	0.606	1.049 (0.689–1.587)	0.824
Any repeat revascularization	286 (9.4)	89 (7.6)	0.069	1.247 (0.983–1.581)	0.069	1.152 (0.859–1.544)	0.346	1.216 (0.925–1.609)	0.161

*SDT* symptom-to-door time, *HR* hazard ratio, *CI* confidence interval, *MACE* major adverse cardiac events, *MI* myocardial infarction, *LVEF* left ventricular ejection fraction, *SBP* systolic blood pressure, *DBP* diastolic blood pressure, *DBT* door-to-balloon time, *EMS* emergency medical service, *PCI* percutaneous coronary intervention, *CK-MB* creatine kinase myocardial band.

*Adjusted by male sex, age, LVEF, SBP, DBP, DBT, atypical chest pain, dyspnea, Q-wave in electrocardiogram, ST-segment depression, T-wave inversion, Killip class II/III, EMS, non-PCI center, hypertension, diabetes mellitus, previous stroke, current smoker, peak CK-MB, peak troponin-I, and blood glucose (Table S3).

**Table 3:** Clinical outcomes between the complex and non-complex groups in patient with or without delayed hospitalization at 3 years.

Outcomes	SDT < 24 h, n = 3149						
	Complex (n = 1464, group A)	Non-complex (n = 1685, group C)	Log-rank	Unadjusted		Multivariable-adjusted*	
				HR (95% CI)	p	HR (95% CI)	p
MACE	244 (16.7)	224 (13.3)	0.009	1.273 (1.064–1.526)	0.009	1.235 (1.016–1.502)	0.034
All-cause death	96 (6.6)	87 (5.2)	0.097	1.278 (0.956–1.708)	0.098	1.038 (0.840–1.540)	0.405
Cardiac death	50 (3.4)	48 (2.9)	0.354	1.206 (0.811–1.792)	0.355	1.110 (0.734–1.678)	0.622
Non-cardiac death	46 (3.2)	39 (2.3)	0.150	1.367 (0.892–2.094)	0.151	1.163 (0.744–1.818)	0.508
Recurrent MI	47 (3.3)	56 (3.4)	0.889	0.973 (0.660–1.434)	0.890	1.007 (0.666–1.523)	0.972
Any repeat revascularization	149 (10.5)	137 (8.3)	0.045	1.267 (1.004–1.597)	0.046	1.323 (1.026–1.705)	0.031

Outcomes	SDT ≥ 24 h, n = 1224						
	Complex (n = 642, group B)	Non-complex (n = 582, group D)	Log-rank	Unadjusted		Multivariable-adjusted*	
				HR (95% CI)	p	HR (95% CI)	p
MACE	114 (17.8)	81 (13.9)	0.066	1.305 (0.981–1.735)	0.067	1.381 (1.016–1.876)	0.039
All-cause death	65 (10.1)	43 (7.4)	0.084	1.402 (0.954–2.061)	0.086	1.606 (1.056–2.442)	0.027
Cardiac death	39 (6.1)	25 (4.3)	0.149	1.445 (0.874–2.387)	0.151	1.732 (1.000–3.000)	0.051
Non-cardiac death	26 (4.0)	18 (3.1)	0.335	1.342 (0.736–2.448)	0.337	1.448 (0.758–2.770)	0.263
Recurrent MI	25 (3.9)	16 (2.8)	0.241	1.453 (0.776–2.721)	0.243	1.355 (0.716–2.566)	0.350
Any repeat revascularization	50 (8.2)	39 (6.9)	0.422	1.187 (0.781–1.804)	0.422	1.147 (0.734–1.794)	0.547

Outcomes	Total, n = 4373						
	Complex (n = 2106, group A + C)	Non-complex (n = 2267, group B + D)	Log-rank	Unadjusted		Multivariable-adjusted*	
				HR (95% CI)	p	HR (95% CI)	p
MACE	358 (17.0)	305 (13.5)	0.001	1.285 (1.103–1.497)	0.001	1.283 (1.089–1.512)	0.003
All-cause death	161(7.6)	130 (5.7)	0.011	1.348 (1.070–1.699)	0.011	1.303 (1.021–1.663)	0.033
Cardiac death	89 (4.3)	73 (3.4)	0.073	1.326 (0.973–1.807)	0.074	1.330 (0.960–1.845)	0.087
Non-cardiac death	72 (3.3)	57 (2.3)	0.070	1.377 (0.972–1.948)	0.071	1.271 (0.881–1.834)	0.199
Recurrent MI	72 (3.5)	72 (3.3)	0.604	1.090 (0.786–1.511)	0.605	1.110 (0.786–1.565)	0.554
Any repeat revascularization	199 (9.8)	176 (8.0)	0.041	1.235 (1.008–1.513)	0.041	1.281 (1.028–1.598)	0.028

*SDT* symptom-to-door time, *HR* hazard ratio, *CI* confidence interval, *MACE* major adverse cardiac events, *MI* myocardial infarction, *LVEF* left ventricular ejection fraction, *DBP* diastolic blood pressure, *DBT* door-to-balloon time, *EMS* emergency medical service, *CK-MB* creatine kinase myocardial band, *HDL* high-density lipoprotein, *GRACE* Global Registry of Acute Coronary Events.

*Adjusted by male sex, age, LVEF, DBP, DBT, atypical chest pain, dyspnea, ST-segment depression, Killip class II/III, EMS, hypertension, diabetes mellitus, current smoker, peak CK-MB, peak troponin-I, blood glucose, total cholesterol, triglyceride, HDL-cholesterol, GRACE risk score (Table S4).

**Table 4:** Independent predictors for all-cause death.

Variables	Complex				Non-complex			
	Unadjusted		Adjusted		Unadjusted		Adjusted	
	HR (95% CI)	p value	HR (95% CI)	p value	HR (95% CI)	p value	HR (95% CI)	p value
SDT < 24 h vs. SDT ≥ 24 h	0.635 (0.463–0.870)	0.005	0.650 (0.491–0.945)	0.007	0.695 (0.482–1.002)	0.051	0.954 (0.651–1.396)	0.807
Male	1.429 (1.036–1.972)	0.030	1.175 (0.837–1.651)	0.352	1.815 (1.273–2.587)	0.001	1.191 (0.824–1.722)	0.353
Age, ≥ 65 years	3.653 (2.469–5.404)	< 0.001	2.290 (1.473–3.525)	< 0.001	5.436 (3.571–7.190)	< 0.001	3.343 (1.950–5.732)	< 0.001
LVEF, < 50%	3.589 (2.627–4.904)	< 0.001	2.471 (1.773–3.443)	< 0.001	3.295 (2.336–4.648)	< 0.001	1.908 (1.324–2.747)	0.001
DBT	1.002 (1.000–1.004)	0.033	1.000 (0.998–1.002)	0.702	1.003 (0.998–1.008)	0.093	1.000 (0.996–1.004)	0.981
Atypical chest pain	3.074 (2.225–4.246)	< 0.001	1.945 (1.349–2.804)	< 0.001	3.711 (2.590–5.317)	< 0.001	2.320 (1.564–3.440)	< 0.001
Dyspnea	1.681 (1.218–2.318)	0.002	1.238 (0.860–1.783)	0.250	0.474 (0.331–0.680)	< 0.001	1.027 (0.683–1.542)	0.900
EMS ( +)	1.035 (0.618–1.735)	0.895	1.063 (0.631–1.791)	0.818	1.652 (1.016–2.688)	0.043	1.439 (1.007–2.418)	0.173
Hypertension	1.434 (1.038–1.980)	0.029	1.043 (0.744–1.463)	0.806	2.028 (1.410–2.916)	< 0.001	1.235 (0.846–1.804)	0.275
Diabetes mellitus	2.044 (1.500–2.784)	< 0.001	1.583 (1.149–2.181)	0.005	1.673 (1.170–2.392)	0.005	1.220 (0.839–1.760)	0.286
GRACE risk score > 140	3.642 (2.625–5.051)	< 0.001	1.786 (1.224–2.607)	0.003	4.124 (2.867–6.185)	< 0.001	3.087 (2.003–4.757)	< 0.001

*HR* hazard ratio, *CI* confidence interval, *SDT* symptom-to-door time, *LVEF* left ventricular ejection fraction, *DBT* door-to-balloon time, *EMS* emergency medical service, *GRACE* Global Registry of Acute Coronary Events.

## Discussion

The main findings of this nonrandomized, multicenter, prospective cohort study were as follows. First, in the complex group and the total study population, all-cause death and CD rates were significantly higher for patients with SDT ≥ 24 h than for those with SDT < 24 h after adjustment. Second, in the non-complex group, all clinical outcomes showed no significant different between patients with SDT < 24 h and those with SDT ≥ 24 h after adjustment. Third, MACE and repeat revascularizations for patients with SDT < 24 h and MACE and all-cause death for patients with SDT ≥ 24 h were significantly higher in the complex group than in the non-complex group. Fourth, in both complex and non-complex groups, old age, a reduced left ventricular ejection fraction, atypical chest pain, and a high GRACE risk score were common independent predictors of all-cause death.

Although prehospital delay is considered an important factor in long-term mortality in patients with STEMI^8^, the importance of prehospital delay for patients with NSTEMI remains unclear. Till date, few studies have shown the effects of delayed hospitalization on the clinical outcomes of patients with NSTEMI^9,10^. As mentioned earlier, Cha et al.^9^ reported that the 3-year all-cause mortality was significantly higher for patients with SDT ≥ 24 h than for those with SDT < 24 h (aHR, 1.35; 95% CI, 1.17–1.56; p < 0.001). A Turkish study reported^11^ that patients with NSTEMI who were transferred from a non-PCI center to a PCI center showed a 60% delay in the total prehospital delay (p < 0.001).

In the present study, patients with SDT ≥ 24 h group were more likely to be older and tended to have a high cardiovascular risk profile (such as a high mean GRACE risk score and higher rates of Killip class II/III and diabetes mellitus) than did patients with < 24 h in both the complex and non-complex groups (Table 1). Interestingly, these characteristics of NSTEMI patients in our study population were similar to those of patients who presented later (between 12 and 24 h after symptom onset) in other studies^3,5,12^. In general, older patients may have difficulty in moving and may need help with transportation, which may contribute to delayed hospitalization^13^. In addition, older patients may have other comorbidities, which may lead to delayed recognition^13^. Delayed hospitalization in diabetes mellitus may arise from inadequate sensory feeling caused by diabetic neuropathy and a higher rate of silent myocardial infarction in this population^13^. In patients with acute coronary syndrome, atypical ischemic symptoms are frequent and lead to delayed hospitalization^14,15^. In our study, old age (≥ 65 years, p < 0.001), diabetes mellitus (p < 0.001), a high GRACE risk score (p < 0.001), and atypical chest pain (p < 0.001) were independent predictors of all-cause death in the complex group (Table 4).

Karwowski et al.^16^ reported that although there is a lack of data in patients with NSTEMI, rapid restoration of flow could result in a smaller infarct size and better prognosis. Therefore, patients with SDT ≥ 24 h had a larger infarct size and poorer prognosis than did those with SDT < 24 h. Compared to PCI for non-complex lesions, PCI for complex lesions typically requires a longer procedural duration and demands a relatively advanced level of procedural skills. Consequently, there is a relatively higher risk of hemodynamic instability^10^. Therefore, in the complex group, considering the similar DBT (p = 0.162) for patients with SDT < 24 h and those with SDT ≥ 24 h (Table 1), the former showed lower all-cause death (aHR, 1.752; p = 0.004) and CD (aHR, 1.966; p = 0.010) rates than did the latter (Table 2). Moreover, the symptom-to-balloon time was significantly lower in the SDT < 24 h group than in the SDT ≥ 24 h group (p < 0.001) (Table 1). However, in the non-complex group, the 3-year mortality rate was not significantly different between the two groups (Table 2). Because of limited data^9,11^ regarding long-term outcomes after new-generation DES implantation in patients with NSTEMI with SDT < 24 h and those with SDT ≥ 24, we could not compare our results with those of other studies; moreover, we could not precisely define the causal relationship in the non-complex group in our study. As reported earlier^10^, we speculate that because PCI for non-complex lesions is a simple technique requiring relatively short procedural times and carrying a low risk of hemodynamic deterioration, the effect of delayed hospitalization on 3-year mortality may be insignificant compared with that in the complex group. However, further studies are required to confirm our results.

A recent study^17^ found that patients requiring complex PCI were older and more frequently showed ACC/AHA type B2/C lesions than did patients not requiring complex PCI. As shown in Table S1 in the Supplementary Appendix, for both patients with SDT < 24 h and SDT ≥ 24 h, the mean age and number of patients with ACC/AHA type B2/C lesions were significantly higher in the complex group than that in the non-complex lesion group. Furthermore, during a 3-year follow-up period, patients who required complex PCI showed a higher rate of target lesion failure (aHR, 1.89; 95% CI, 1.31–2.73; p = 0.001) than did those not requiring complex PCI^17^. Riku et al.^18^ showed that the repeat revascularization rate was significantly higher in the complex group than in the non-complex group (log-rank p = 0.001) during a 10-year follow-up period after sirolimus-eluting stent implantation. Another study^19^ suggested that PCI for complex lesions was independently associated with a higher 2-year MACE rate (aHR, 1.56; p < 0.00001). In the present study, for patients with SDT < 24 h and the total study population, the repeat revascularization rates (aHR, 1.323; p = 0.031 and aHR, 1.281; p = 0.028, respectively) were significantly higher in the complex group than in the non-complex group (Table 3); this was related to a higher MACE rate for both patients with SDT < 24 h and the total study population (aHR, 1.235; p = 0.034 and aHR, 1.283; p = 0.03, respectively). Patients who underwent PCI for unprotected left main coronary disease (LM) showed a higher rate of 1-year all-cause death (5.6% vs. 2.3%; p < 0.001) than did patients who underwent PCI for non-LM^20^. During a 10-year follow-up period, the all-cause death rate was 2.9 times higher in patients with multivessel disease than for those with one-vessel disease^21^. A previous study^22^ showed that a stepwise increase in MACE (composite of death, MI, and target lesion revascularization (TLR)) with an increasing stent length (8.0%, 10.1%, 11.8%, and 14.8%, p < 0.001). In our study, all-cause death rates were significantly higher in the complex group than that in the non-complex group for both patients with SDT ≥ 24 h and total study population (aHR, 1.606; p = 0.027 and aHR, 1.303; p = 0.033, respectively) (Table 3).

Although we tend to assume that the long-term clinical outcomes could be poorer for patients with the complex lesions than for patents with non-complex group after PCI^10^, there are no data showing the different effects of delayed hospitalization on long-term clinical outcomes in these patients. From the Korea Acute Myocardial Infarction Registry-National Institute of Health (KAMIR-NIH), a conclusion could not be reached because of an insufficient sample size. However, 20 tertiary high-volume university hospitals participated in this study, and we believe that our results could provide useful information for interventional cardiologists with regard to the importance of shortening SDT, especially for patients with NSTEMI and complex lesions. Furthermore, based on the findings from Table 4, which indicate that complex lesions and delayed hospitalization are associated with a higher mortality rate in NSTEMI patients with old age, diabetes mellitus, a high GRACE risk score, and atypical chest pain, it is evident that increased attention, more proactive follow-up, and guideline-directed intensive treatment are warranted^23^. Therefore, we believe that our findings can contribute to a decrease in the mortality rate for patients who present with NSTEMI and complex lesions, particularly those with delayed hospitalization.

This study has some limitations. First, the KAMIR-NIH data concerning transfers, distance to the nearest hospital, and the presence or absence of large differences between hospitals in the percentage of patients with delayed hospitalization were not mandatory variables; therefore we could not include them in our analysis. We believe that this is a major limitation. Second, although bifurcation lesions and chronic total occlusion lesions could be included in the complex lesion group^24,25^, information regarding these variables was not available in KAMIR-NIH. Third, regarding the characteristics of the registry data, there may have been some underreported and/or missing data. Fourth, although we performed multivariable-adjusted and PS-adjusted analyses to strengthen our results, variables not included in the KAMIR-NIH study may have affected study outcomes. Fifth, because the primary and secondary outcomes were compared based on the 24 h cutoff point, the results could be altered according to different cutoff points of delayed hospitalization. Sixth, some subgroups had relatively small sample sizes; hence, their analyses may have been underpowered for the detection of clinically meaningful differences. Seventh, although the total procedure time^21^, total amount of radiation^26^, and total doses of contrast media^27^ during the procedure were important in the complex groups, we could not include these variables in this analysis owing to the limitations of the KAMIR-NIH data. Eighth, although previous reports^28,29^ showed a relationship between procedural characteristics and operator volume during PCI, the operator volume according to individual participating centers was not included in this analysis; this variable may have acted as an important source of bias in this study. Finally, the 3-year follow-up period in this study was relatively short for estimating long-term clinical outcomes.

In conclusion, this multicenter, prospective cohort study showed that among NSTEMI patients with complex lesions, delayed hospitalization was associated with a higher 3-year mortality rate. Thus, our results emphasize the importance of SDT, especially for patients with complex lesions. In addition, old age, diabetes mellitus, a high GRACE risk score, and atypical chest pain were independent predictors of all-cause death in the complex group. Therefore, patients with these factors require increased attention, proactive follow-up, and guideline-directed intensive treatment. Further large-scale and long-term follow-up studies are needed to confirm our results.

## Methods

### Study population

In total, 13,104 patients with AMI were recruited from KAMIR-NIH^30^ between November 2011 and December 2015. Twenty high-volume PCI centers in the Republic of Korea participated in the KAMIR-NIH study. At the time of initial enrollment, only patients aged ≥ 18 years were included. We excluded patients who did not undergo PCI (n = 1369, 10.4%), those who underwent balloon angioplasty (n = 739, 5.6%), unsuccessful PCI (n = 152, 1.2%), coronary artery bypass graft (CABG, n = 44, 0.4%), bare-metal stents, or first-generation (1G)-DES implantation (n = 708, 5.4%); those with STEMI (n = 5365, 40.9%); those who were lost to follow-up (n = 134, 1.0%); and those with cardiogenic shock, cardiopulmonary resuscitation on admission; and in-hospital death (n = 220) (Fig. 1). Overall, 43,733 patients with NSTEMI who underwent successful PCI using new-generation DES were enrolled and divided into complex (n = 2106, 48.2%) and non-complex (n = 2267, 51.8%). In both groups, patients were subdivided according to SDT < 24 h (group A [n = 1464] and group C [n = 1685]) and SDT ≥ 24 h (group B [n = 642] and group D [n = 582]) (Fig. 1). The types of new-generation DES used during PCI are shown in the footnotes of Table 1. According to the ethical guidelines of the 2004 Declaration of Helsinki, this study was approved by the Ethics Committee of each participating center and the Chonnam National University Hospital Institutional Review Board Ethics Committee (CNUH-2011–172). A total of 4573 patients who were included in the study provided written informed consent prior to enrollment. They completed a 3-year clinical follow-up through face-to-face interviews, phone calls, and chart reviews. Enrolled data were collected from all participating PCI centers using a web-based system. Event adjudication processes have been described in a previous publication by KAMIR investigators^30^. This study was performed using a web-based report from the Internet-based Clinical Research and Trial management system, supported by a grant from the Korean Centers for Disease Control and Prevention since November 2011.

### PCI procedure and medical treatment

The operators performed CAG and PCI via a transfemoral or transradial approach in accordance with general guidelines^31^. The patients were prescribed 200–300 mg aspirin, 300–600 mg clopidogrel, 180 mg ticagrelor, and 60 mg prasugrel as loading doses before PCI. After PCI, 100 mg aspirin was recommended for all patients, combined with 75 mg clopidogrel once daily, 90 mg ticagrelor twice daily, or 5–10 mg prasugrel once daily for a minimum of one year. Individual operators were able to choose the access site, revascularization strategy, and DES without any restrictions.

### Study definitions and clinical outcomes

We defined NSTEMI based on the fourth universal definition of MI^32^. Successful PCI was defined as residual stenosis of < 30% and thrombolysis in MI (TIMI) flow grade 3 in the IRA. We calculated the GRACE risk score^33^ for all enrolled patients. Complex lesions during PCI were defined per the following criteria: PCI for LM, multivessel PCI (≥ 2 major epicardial coronary vessels treated in one PCI session), multiple stent implantation (≥ 3 stents per patient), or a total deployed stent length greater than 38 mm^24,25^. The primary outcome was the rate of MACE defined as all-cause death, recurrent MI, and any repeat revascularization, at 3 years, while the secondary outcomes were individual MACE components. Any repeat coronary revascularization included target-lesion revascularization, target-vessel revascularization (TVR), and non-TVR. All-cause death was considered cardiac death (CD) unless an undisputed noncardiac cause was present^34^. Previously, we reported definitions of re-MI, TLR, TVR, and non-TVR^35^.

### Statistical analysis

For continuous variables, between-group differences were evaluated using unpaired t-tests. Data are expressed as mean ± standard deviation or median (interquartile range). For discrete variables, between-group differences were expressed as counts and percentages and were analyzed using the chi-square or Fisher’s exact test. We performed univariate analyses for all variables in the groups with or without delayed hospitalization and the groups with or without complex lesions; a p-value of < 0.005 was considered statistical significance. Subsequently, a multicollinearity test^36^ was performed for the included variables to confirm the noncollinearity among them (Table S3 in the Supplementary Appendix). We measured the variance inflation factor values to determine the degree of multicollinearity among the variables. A measured variance inflation factor > 5 was considered as high correlation^37^. Multicollinearity was presumed when the tolerance value was < 0.1^38^ or the condition index was > 10^37^. Finally, the following variables were included in the multivariate Cox regression analysis: male sex, age, LVEF, systolic blood pressure, diastolic blood pressure, DBT, atypical chest pain, dyspnea, Q-wave on electrocardiogram, ST-segment depression, T-wave inversion, Killip class II/III, EMS, non-PCI center, hypertension, diabetes mellitus, previous stroke, current smoker, peak CK-MB, peak troponin-I, and blood glucose. Moreover, to correct for confounding variables, a PS-adjusted analysis was performed using a logistic regression model. All the baseline characteristics shown in Table 1 were included in the PS-adjusted analysis. The c-statistic for the PS-matched (PSM) analysis in this study was 0.703. Using the nearest available pair-matching method in a 1:1 fashion, patients in the SDT ≥ 24 h group were matched to those in the SDT < 24 h group. The caliper width was 0.01. Table S3 shows the results of the collinearity test for MACE between the SDT < 24 h and SDT ≥ 24 h groups. Various clinical outcomes were estimated using Kaplan–Meier curve analysis, and group differences were compared using the log-rank test. A p-value of < 0.05 was considered statistically significant. Table S4 in the Supplementary Appendix shows the results of the collinearity test for MACE between the complex and non-complex groups. SPSS software version 20 (IBM, Armonk, NY, USA) was used to perform the statistical analyses.

### Supplementary Information

Supplementary Tables.

## Data Availability

Data is contained with the article or supplementary material.
