# Supplementary material for: Effect of delayed hospitalization on patients with non-ST-segment elevation myocardial infarction and complex lesions undergoing successful new-generation drug-eluting stents implantation
Source: Sci Rep. 2023 Sep 26;13:16067. doi: 10.1038/s41598-023-43385-3 (PMC10522700; doi:10.1038/s41598-023-43385-3)
Supplement: Supplementary file 1 — Supplementary Tables. [file 41598_2023_43385_MOESM1_ESM.docx]

**Supplementary Appendix**

**Effect of delayed hospitalization in patients with non-ST-segment elevation myocardial infarction and complex lesions undergoing successful new-generation drug-eluting stents implantation**

**Short title:** Prehospital delay in NSTEMI and complex lesions

Yong Hoon Kim^1,8^, Ae-Young Her^1,8^, Seung-Woon Rha^2^, Cheol Ung Choi^2^, Byoung Geol Choi^3^, Ji Bak Kim^2^, Soohyung Park^2^, Dong Oh Kang^2^, Ji Young Park^4^, Woong Gil Choi^5^, Sang-Ho Park^6^ & Myung Ho Jeong^7^

**Supplementary Online Contents**

**Table S1.** Baseline characteristics between the complex and non-complex groups according to the presence or absence of delayed hospitalization.

**Table S2.** Baseline characteristics between the SDT <24 h and SDT≥24 h groups in the total study population and propensity score-matched patients.

**Table S3** Results of the collinearity test for MACE between the SDT <24 h and SDT ≥24 h groups

**Table S4.** Results of the collinearity test for MACE between the complex and non-complex groups

| Variables | SDT <24 h (n = 3149) | | |  | SDT ≥24 h (n = 1224) | | |
| --- | --- | --- | --- | --- | --- | --- | --- |
|  | Complex  (n = 1464, group A) | Non-complex  (n = 1685, group C) | p value |  | Complex  (n = 642, group B) | Non-complex  (n = 582, group D) | p value |
| Male, n (%) | 1082 (73.9) | 1295 (76.9) | 0.056 |  | 422 (65.7) | 399 (68.6) | 0.301 |
| Age, years | 64.6 ± 11.6 | 61.8 ± 12.1 | <0.001 |  | 67.2 ± 11.5 | 65.3 ± 12.2 | 0.004 |
| LVEF, % | 54.3 ± 10.4 | 55.6 ± 9.5 | <0.001 |  | 53.0 ± 11.2 | 54.7 ± 11.2 | 0.011 |
| BMI, kg/m^2^ | 24.2 ± 3.3 | 24.2 ± 3.3 | 0.967 |  | 24.0 ± 3.7 | 24.0 ± 3.2 | 0.966 |
| SBP, mmHg | 138.2 ± 26.2 | 137.8 ± 25.6 | 0.632 |  | 134.6 ± 23.5 | 134.8 ± 23.1 | 0.901 |
| DBP, mmHg | 81.9 ± 14.9 | 83.2 ± 15.5 | 0.022 |  | 80.1 ± 14.0 | 81.7 ± 13.4 | 0.050 |
| SDT, hours | 4.0 (1.8-8.6) | 3.8 (1.6-8.2) | 0.397 |  | 72.0 (35.6-161.4) | 71.4 (33.6-120.0) | 0.231 |
| DBT, hours | 14.9 (4.1-26.7) | 12.2 (3.9-23.9) | 0.001 |  | 16.4 (4.0-24.9) | 16.9 (4.0-27.1) | 0.531 |
| Atypical chest pain, n (%) | 185 (12.6) | 187 (11.1) | 0.185 |  | 149 (23.2) | 117 (20.1) | 0.212 |
| Dyspnea, n (%) | 341 (23.3) | 327 (19.4) | 0.009 |  | 199 (31.0) | 151 (25.9) | 0.057 |
| EKG on admission |  |  |  |  |  |  |  |
| Q-wave, n (%) | 103 (7.0) | 112 (6.6) | 0.672 |  | 69 (10.7) | 58 (10.0) | 0.708 |
| ST-segment depression, n (%) | 374 (25.5) | 350 (20.8) | 0.002 |  | 128 (19.9) | 98 (16.8) | 0.184 |
| T-wave inversion, n (%) | 336 (23.0) | 349 (20.7) | 0.130 |  | 175 (27.3) | 166 (28.5) | 0.655 |
| Atrial fibrillation, n (%) | 53 (3.6) | 60 (3.6) | 0.929 |  | 28 (4.4) | 18 (3.1) | 0.293 |
| Killip class 1I/III, n (%) | 240 (16.4) | 180 (10.7) | <0.001 |  | 137 (21.3) | 84 (14.4) | 0.002 |
| First medical contact |  |  |  |  |  |  |  |
| EMS, n (%) | 176 (12.0) | 203 (12.0) | 0.982 |  | 27 (4.2) | 15 (2.6) | 0.158 |
| Non-PCI center, n (%) | 752 (51.4) | 838 (49.7) | 0.372 |  | 380 (59.2) | 338 (58.1) | 0.727 |
| PCI center, n (%) | 536 (36.6) | 644 (38.2) | 0.356 |  | 235 (36.6) | 229 (39.3) | 0.345 |
| Hypertension, n (%) | 798 (54.5) | 821 (48.7) | 0.001 |  | 388 (60.4) | 304 (52.2) | 0.004 |
| Diabetes mellitus, n (%) | 472 (32.2) | 413 (24.5) | <0.001 |  | 251 (39.1) | 168 (28.9) | <0.001 |
| Dyslipidemia, n (%) | 179 (12.2) | 208 (12.3) | 0.957 |  | 75 (11.7) | 67 (11.5) | 0.929 |
| Previous MI, n (%) | 98 (6.7) | 115 (6.8) | 0.943 |  | 46 (7.2) | 40 (6.9) | 0.911 |
| Previous PCI, n (%) | 146 (10.0) | 178 (10.6) | 0.957 |  | 60 (9.3) | 51 (8.8) | 0.723 |
| Previous CABG, n (%) | 10 (0.7) | 11 (0.7) | 0.917 |  | 5 (0.8) | 6 (1.0) | 0.765 |
| Previous HF, n (%) | 24 (1.6) | 18 (1.1) | 0.212 |  | 11 (1.7) | 8 (1.4) | 0.653 |
| Previous stroke, n (%) | 80 (5.5) | 80 (4.7) | 0.372 |  | 52 (8.1) | 34 (5.8) | 0.145 |
| Current smokers, n (%) | 518 (35.4) | 694 (41.2) | 0.001 |  | 170 (26.5) | 204 (35.1) | 0.001 |
| Peak CK-MB, mg/dL | 24.1 (7.2-83.0) | 27.1 (6.7-95.3) | 0.031 |  | 11.4 (4.8-33.5) | 11.3 (4.1-42.6) | 0.967 |
| Peak troponin-I, ng/mL | 7.2 (1.6-23.6) | 11.0 (2.0-23.0) | 0.386 |  | 4.9 (1.5-13.7) | 4.0 (0.9-15.3) | 0.299 |
| Blood glucose, mg/dL | 165.8 ± 83.1 | 152.5 ± 67.3 | <0.001 |  | 153.6 ± 58.8 | 145.0 ± 75.8 | 0.039 |
| Serum creatinine (mg/L) | 1.13 ± 1.20 | 1.07 ± 1.30 | 0.198 |  | 1.21 ± 1.31 | 1.11 ± 1.35 | 0.187 |
| Total cholesterol, mg/dL | 179.0 ± 44.0 | 182.8 ± 44.2 | 0.017 |  | 176.3 ± 44.2 | 177.3 ± 45.1 | 0.696 |
| Triglyceride, mg/L | 130.0 ± 106.7 | 139.0 ± 129.2 | 0.033 |  | 128.8 ± 107.6 | 128.6 ± 83.9 | 0.952 |
| HDL cholesterol, mg/L | 42.5 ± 11.6 | 43.8 ± 11.1 | 0.001 |  | 41.5 ± 11.3 | 42.6 ± 12.1 | 0.129 |
| LDL cholesterol, mg/L | 113.2 ± 38.6 | 115.7 ± 36.0 | 0.061 |  | 111.5 ± 36.5 | 111.9 ± 38.8 | 0.883 |
| GRACE risk score | 131.1 ± 38.3 | 120.6 ± 35.6 | <0.001 |  | 135.4 ± 35.2 | 127.4 ± 32.5 | <0.001 |
| Discharge medications, n (%) |  |  |  |  |  |  |  |
| Aspirin, n (%) | 1455 (99.4) | 1676 (99.5) | 0.816 |  | 636 (99.1) | 574 (98.6) | 0.593 |
| Clopidogrel, n (%) | 1020 (69.7) | 1210 (71.8) | 0.195 |  | 469 (73.1) | 441 (75.8) | 0.295 |
| Ticagrelor, n (%) | 301 (20.6) | 323 (19.2) | 0.347 |  | 116 (18.1) | 88 (15.1) | 0.192 |
| Prasugrel, n (%) | 143 (9.8) | 152 (9.0) | 0.500 |  | 57 (8.9) | 53 (9.1) | 0.920 |
| BBs, n (%) | 1274 (87.0) | 1439 (85.4) | 0.196 |  | 552 (86.0) | 486 (83.5) | 0.233 |
| ACEI or ARBs, n (%) | 1218 (83.2) | 1416 (84.0) | 0.530 |  | 527 (82.1) | 478 (82.1) | 0.984 |
| Statin, n (%) | 1401 (95.7) | 1613 (95.7) | 0.967 |  | 609 (94.9) | 555 (95.4) | 0.694 |
| Anticoagulant, n (%) | 20 (1.4) | 30 (1.8) | 0.393 |  | 21 (3.3) | 15 (2.6) | 0.503 |
| Infarct-related artery |  |  |  |  |  |  |  |
| Left main, n (%) | 80 (5.5) | - | - |  | 45 (7.0) | - | - |
| LAD, n (%) | 598 (40.8) | 748 (44.4) | 0.047 |  | 259 (40.3) | 257 (44.2) | 0.183 |
| LCx, n (%) | 341 (23.3) | 502 (29.8) | <0.001 |  | 120 (18.7) | 161 (27.7) | <0.001 |
| RCA, n (%) | 445 (30.4) | 435 (25.8) | 0.005 |  | 218 (34.0) | 164 (28.2) | 0.031 |
| Treated vessel |  |  |  |  |  |  |  |
| Left main, n (%) | 122 (8.3) | - | - |  | 73 (11.4) | - | - |
| LAD, n (%) | 1038 (70.9) | 748 (44.4) | <0.001 |  | 466 (72.6) | 257 (44.2) | <0.001 |
| LCx, n (%) | 724 (49.5) | 502 (29.8) | <0.001 |  | 304 (47.4) | 161 (27.7) | <0.001 |
| RCA, n (%) | 716 (48.9) | 435 (25.8) | <0.001 |  | 329 (51.2) | 164 (28.2) | <0.001 |
| ACC/AHA type B2/C lesions, n (%) | 1293 (88.3) | 1348 (80.0) | <0.001 |  | 575 (89.6) | 453 (77.8) | <0.001 |
| Pre-PCI TIMI flow grade 0/1, n (%) | 563 (38.5) | 646 (38.3) | 0.971 |  | 256 (39.9) | 207 (35.6) | 0.125 |
| GP IIb/IIIa inhibitor, n (%) | 140 (9.6) | 127 (7.5) | 0.047 |  | 61 (9.5) | 44 (7.6) | 0.261 |
| Transradial approach, n (%) | 717 (49.0) | 916 (54.4) | 0.003 |  | 345 (53.7) | 350 (60.1) | 0.024 |
| IVUS/OCT, n (%) | 441 (28.1) | 385 (22.8) | 0.001 |  | 177 (27.6) | 138 (23.7) | 0.132 |
| FFR, n (%) | 41 (2.8) | 33 (2.0) | 0.126 |  | 19 (3.0) | 7 (1.2) | 0.045 |
| Drug-eluting stents^a^ |  |  |  |  |  |  |  |
| ZES, n (%) | 329 (22.5) | 426 (25.3) | 0.066 |  | 123 (19.2) | 126 (21.6) | 0.287 |
| EES, n (%) | 871 (59.5) | 773 (45.9) | <0.001 |  | 399 (62.1) | 254 (43.6) | <0.001 |
| BES, n (%) | 203 (13.9) | 427 (25.3) | <0.001 |  | 98 (15.3) | 181 (31.1) | <0.001 |
| Others, n (%) | 61 (4.2) | 59 (3.5) | 0.351 |  | 22 (3.4) | 21 (3.6) | 0.878 |
| Stent diameter (mm) | 3.06 ± 0.40 | 3.10 ± 0.44 | <0.001 |  | 3.04 ± 0.40 | 3.10 ± 0.44 | 0.004 |
| Stent length (mm) | 36.8 ± 15.9 | 22.9 ± 6.09 | <0.001 |  | 37.3 ± 16.5 | 21.8 ± 5.93 | <0.001 |
| Number of stents | 1.40 ± 0.57 | 1.03 ± 0.19 | <0.001 |  | 1.38 ± 0.56 | 1.02 ± 0.18 | <0.001 |

**Table S1.** Baseline characteristics between the complex and non-complex groups according to the presence or absence of delayed hospitalization. Values are means ± standard deviation or median (interquartile range) or numbers and percentages. The p values for continuous data were obtained from the unpaired t-test. The p values for categorical data from chi-square or Fisher’s exact test. *SDT* symptom-to-door time, *LVEF* left ventricular ejection fraction, *BMI* body mass index, *SBP* systolic blood pressure, *DBP,* diastolic blood pressure, *DBT* door-to-balloon time, *EKG* electrocardiogram, *EMS* emergency medical service, *PCI* percutaneous coronary intervention, *MI* myocardial infarction, *CABG* coronary artery bypass graft, *HF* heart failure, *CK-MB* creatine kinase myocardial band, *HDL* high-density lipoprotein, *LDL* low-density lipoprotein, *GRACE* Global Registry of Acute Coronary Events, *BBs* ß-blockers, *ACEIs* angiotensin-converting enzyme inhibitors, *ARBs* angiotensin receptor blockers, *LAD* left anterior descending artery, *LCx* left circumflex artery, *RCA* right coronary artery, *ACC/AHA* American College of Cardiology/American Heart Association, *TIMI* thrombolysis in myocardial infarction, *GP* glycoprotein, *IVUS* intravascular ultrasound, *OCT* optical coherence tomography, *FFR* fractional flow reserve, *ZES* zotarolimus-eluting stent, *EES* everolimus-eluting stent, *BES* biolimus-eluting stent. ^a^ Drug-eluting stents were composed of ZES (Resolute integrity stent; Medtronic, Inc., Minneapolis, MN), EES (Xience Prime stent, Abbott Vascular, Santa Clara, CA; or Promus Element stent, Boston Scientific, Natick, MA), and BES (BioMatrix Flex stent, Biosensors International, Morges, Switzerland; or Nobori stent, Terumo Corporation, Tokyo, Japan).

| Variables | All patients (n = 4373) | | |  | Propensity score-matched patients (n = 2267) | | |  |
| --- | --- | --- | --- | --- | --- | --- | --- | --- |
|  | SDT <24 h  (n = 3149, group A+C) | SDT ≥24 h  (n = 1224, group B+D) | p value |  | SDT <24 h  (n = 1112) | SDT ≥24 h  (n = 1112) | p value | SD |
| Male, n (%) | 2377 (75.5) | 821 (67.1) | <0.001 |  | 744 (66.9) | 770 (69.2) | 0.255 | -0.49 |
| Age, years | 63.1 ± 12.0 | 66.3 ± 11.9 | <0.001 |  | 65.7 ± 11.6 | 65.6 ± 11.9 | 0.714 | 0.16 |
| LVEF, % | 55.0 ± 9.9 | 53.8 ± 11.1 | 0.001 |  | 54.6 ± 10.6 | 54.5 ± 10.8 | 0.802 | 0.10 |
| BMI, kg/m^2^ | 24.2 ± 3.3 | 24.0 ± 3.3 | 0.184 |  | 23.9 ± 3.2 | 24.1 ± 3.2 | 0.294 | -0.45 |
| SBP, mmHg | 138.0 ± 25.9 | 134.7 ± 23.3 | <0.001 |  | 134.9 ± 23.6 | 134.9 ± 23.4 | 0.988 | 0.01 |
| DBP, mmHg | 82.6 ± 15.2 | 80.8 ± 13.7 | <0.001 |  | 81.1 ± 14.0 | 81.0 ± 13.8 | 0.937 | 0.02 |
| DBT, hours | 13.5 (4.0-25.1) | 16.7 (4.0-25.7) | 0.003 |  | 14.5 (4.1-25.8) | 16.2 (3.9-25.4) | 0.448 | -0.30 |
| Atypical chest pain, n (%) | 372 (11.8) | 266 (21.7) | <0.001 |  | 199 (17.9) | 201 (18.1) | 0.956 | -0.05 |
| Dyspnea, n (%) | 688 (21.2) | 350 (28.6) | <0.001 |  | 274 (24.6) | 287 (25.8) | 0.558 | -0.28 |
| EKG on admission |  |  |  |  |  |  |  |  |
| Q-wave, n (%) | 215 (6.8) | 127 (10.4) | <0.001 |  | 107 (9.6) | 106 (9.5) | 0.943 | 0.03 |
| ST-segment depression, n (%) | 724 (23.0) | 226 (18.5) | 0.001 |  | 200 (18.0) | 208 (18.7) | 0.701 | -0.18 |
| T-wave inversion, n (%) | 685 (21.8) | 341 (27.9) | <0.001 |  | 304 (27.3) | 306 (27.5) | 0.962 | -0.04 |
| Atrial fibrillation, n (%) | 113 (3.6) | 46 (3.8) | 0.788 |  | 39 (3.5) | 41 (3.7) | 0.909 | -0.11 |
| Killip class 1I/III, n (%) | 420 (13.3) | 221 (18.1) | <0.001 |  | 175 (15.7) | 184 (16.5) | 0.645 | -0.22 |
| First medical contact |  |  |  |  |  |  |  |  |
| EMS, n (%) | 379 (12.0) | 42 (3.4) | <0.001 |  | 41 (3.7) | 42 (3.9) | 0.911 | -0.10 |
| Non-PCI center, n (%) | 1590 (50.5) | 718 (58.7) | <0.001 |  | 649 (58.4) | 646 (58.1) | 0.931 | 0.06 |
| PCI center, n (%) | 1180 (37.5) | 464 (37.9) | 0.808 |  | 422 (37.9) | 424 (38.1) | 0.965 | -0.04 |
| Hypertension, n (%) | 1619 (51.4) | 692 (56.5) | 0.002 |  | 602 (54.1) | 618 (55.6) | 0.523 | -0.30 |
| Diabetes mellitus, n (%) | 885 (28.1) | 419 (34.2) | <0.001 |  | 364 (32.7) | 354 (31.8) | 0.683 | 0.19 |
| Dyslipidemia, n (%) | 387 (12.3) | 142 (11.6) | 0.570 |  | 141 (12.7) | 132 (11.9) | 0.605 | 0.24 |
| Previous MI, n (%) | 213 (6.8) | 86 (7.0) | 0.790 |  | 76 (6.8) | 75 (6.7) | 0.933 | 0.04 |
| Previous PCI, n (%) | 324 (10.3) | 111 (9.1) | 0.238 |  | 103 (9.3) | 101 (9.1) | 0.941 | 0.07 |
| Previous CABG, n (%) | 21 (0.7) | 11 (0.9) | 0.431 |  | 12 (1.1) | 10 (0.9) | 0.831 | 0.20 |
| Previous HF, n (%) | 42 (1.3) | 19 (1.6) | 0.568 |  | 15 (1.3) | 14 (1.3) | 0.852 | 0.08 |
| Previous stroke, n (%) | 160 (5.1) | 86 (7.0) | 0.016 |  | 82 (7.4) | 68 (6.1) | 0.272 | 0.52 |
| Current smokers, n (%) | 1212 (38.5) | 374 (30.6) | <0.001 |  | 346 (31.1) | 360 (32.4) | 0.554 | -0.27 |
| Peak CK-MB, mg/dL | 25.8 (6.9-88.9) | 11.6 (4.5-37.6) | <0.001 |  | 16.6 (5.2-55.4) | 12.4 (4.7-40.3) | 0.055 | 0.81 |
| Peak troponin-I, ng/mL | 9.9 (2.0-23.0) | 4.9 (1.3-13.7) | <0.001 |  | 5.2 (1.2-22.4) | 5.3 (1.3-14.0) | 0.492 | -0.43 |
| Blood glucose, mg/dL | 158.6 ± 74.8 | 149.6 ± 71.4 | <0.001 |  | 151.1 ± 66.2 | 149.8 ± 72.7 | 0.671 | 0.12 |
| Serum creatinine (mg/L) | 1.10 ± 1.26 | 1.16 ± 1.33 | 0.158 |  | 1.11 ± 1.33 | 1.16 ± 1.36 | 0.395 | -0.36 |
| Total cholesterol, mg/dL | 181.0 ± 43.9 | 176.7 ± 44.3 | 0.004 |  | 176.4 ± 44.0 | 177.3 ± 43.9 | 0.616 | -0.21 |
| Triglyceride, mg/L | 134.9 ± 118.1 | 128.7 ± 95.4 | 0.072 |  | 126.9 ± 91.9 | 129.9 ± 98.5 | 0.452 | -0.32 |
| HDL cholesterol, mg/L | 43.2 ± 11.3 | 42.0 ± 11.5 | 0.002 |  | 42.3 ± 11.2 | 42.3 ± 11.5 | 0.966 | 0.01 |
| LDL cholesterol, mg/L | 114.5 ± 36.3 | 111.6 ± 36.0 | 0.017 |  | 111.9 ± 35.4 | 112.1 ± 36.0 | 0.872 | -0.07 |
| GRACE risk score | 125.5 ± 37.2 | 131.6 ± 34.2 | <0.001 |  | 129.8 ± 36.4 | 129.8 ± 33.9 | 0.978 | 0.01 |
| Discharge medications, n (%) |  |  |  |  |  |  |  |  |
| Aspirin, n (%) | 3131 (99.4) | 1210 (98.9) | 0.073 |  | 1100 (98.9) | 1101 (99.0) | 0.834 | -0.10 |
| Clopidogrel, n (%) | 2230 (70.8) | 910 (74.3) | 0.020 |  | 801 (72.0) | 819 (73.7) | 0.418 | -0.38 |
| Ticagrelor, n (%) | 624 (19.8) | 204 (16.7) | 0.018 |  | 214 (19.2) | 190 (17.1) | 0.206 | 0.55 |
| Prasugrel, n (%) | 295 (9.4) | 110 (9.0) | 0.728 |  | 97 (8.7) | 103 (9.3) | 0.711 | -0.20 |
| BBs, n (%) | 2713 (86.2) | 1038 (84.8) | 0.267 |  | 954 (85.8) | 947 (85.2) | 0.718 | 0.17 |
| ACEI or ARBs, n (%) | 2634 (83.6) | 1005 (82.1) | 0.224 |  | 905 (81.4) | 914 (82.0) | 0.660 | -0.16 |
| Statin, n (%) | 3014 (95.7) | 1164 (95.1) | 0.370 |  | 1062 (95.5) | 1055 (94.9) | 0.552 | 0.28 |
| Anticoagulant, n (%) | 50 (1.6) | 36 (2.9) | 0.005 |  | 28 (2.5) | 27 (2.4) | 0.891 | 0.06 |
| Infarct-related artery |  |  |  |  |  |  |  |  |
| Left main, n (%) | 80 (2.5) | 45 (3.7) | 0.054 |  | - | - | - |  |
| LAD, n (%) | 1346 (42.7) | 516 (42.2) | 0.733 |  | 444 (39.8) | 467 (41.9) | 0.229 | -0.43 |
| LCx, n (%) | 843 (26.8) | 281 (23.0) | 0.010 |  | 269 (24.2) | 264 (23.7) | 0.804 | 0.12 |
| RCA, n (%) | 880 (27.9) | 382 (31.2) | 0.034 |  | 399 (35.8) | 381 (34.3) | 0.450 | 0.31 |
| Treated vessel |  |  |  |  |  |  |  |  |
| Left main, n (%) | 122 (3.9) | 73 (6.0) | 0.003 |  | - | - | - |  |
| LAD, n (%) | 1783 (56.6) | 723 (59.1) | 0.143 |  | 628 (56.5) | 654 (58.8) | 0.283 | -0.47 |
| LCx, n (%) | 1226 (38.9) | 465 (38.0) | 0.580 |  | 430 (38.7) | 421 (37.9) | 0.727 | 0.16 |
| RCA, n (%) | 1151 (38.6) | 493 (40.3) | 0.024 |  | 450 (40.5) | 438 (39.4) | 0.634 | 0.22 |
| ACC/AHA type B2/C lesions, n (%) | 2641 (83.9) | 1028 (84.0) | 0.963 |  | 934 (84.0) | 931 (83.7) | 0.908 | 0.08 |
| Pre-PCI TIMI flow grade 0/1, n (%) | 1209 (38.4) | 463 (37.8) | 0.755 |  | 396 (35.6) | 409 (36.8) | 0.596 | -0.25 |
| GP IIb/IIIa inhibitor, n (%) | 267 (8.5) | 105 (8.6) | 0.904 |  | 100 (9.0) | 89 (8.0) | 0.447 | 0.36 |
| Transradial approach, n (%) | 1633 (51.9) | 695 (56.8) | 0.004 |  | 615 (55.3) | 618 (55.6) | 0.932 | -0.06 |
| IVUS/OCT, n (%) | 796 (25.3) | 315 (25.7) | 0.757 |  | 274 (24.6) | 284 (25.5) | 0.660 | -0.20 |
| FFR, n (%) | 74 (2.3) | 26 (2.1) | 0.736 |  | 28 (2.5) | 26 (2.3) | 0.891 | 0.13 |
| Drug-eluting stents^*^ |  |  |  |  |  |  |  |  |
| ZES, n (%) | 755 (24.0) | 249 (20.3) | 0.010 |  | 227 (20.4) | 228 (20.5) | 0.958 | -0.02 |
| EES, n (%) | 1644 (52.2) | 653 (53.3) | 0.500 |  | 585 (52.6) | 584 (52.5) | 0.966 | 0.02 |
| BES, n (%) | 630 (20.0) | 279 (22.8) | 0.042 |  | 266 (23.9) | 260 (23.4) | 0.765 | 0.12 |
| Others, n (%) | 120 (3.8) | 43 (3.5) | 0.722 |  | 34 (3.1) | 40 (3.6) | 0.555 | -0.28 |
| Stent diameter (mm) | 3.08 ± 0.42 | 3.07 ± 0.42 | 0.399 |  | 3.07 ± 0.41 | 3.07 ± 0.42 | 0.848 | 0.21 |
| Stent length (mm) | 29.4 ± 13.6 | 29.9 ± 14.8 | 0.288 |  | 29.2 ± 13.7 | 29.7 ± 14.7 | 0.342 | -0.40 |
| Number of stents | 1.20 ± 0.45 | 1.21 ± 0.46 | 0.430 |  | 1.19 ± 0.43 | 1.21 ± 0.46 | 0.181 | -0.45 |

**Table S2.** Baseline characteristics between the SDT <24 h and SDT≥24 h groups in the total study population and propensity score-matched patients. Values are means ± standard deviation or median (interquartile range) or numbers and percentages. The p values for continuous data were obtained from the unpaired t-test. The p values for categorical data from chi-square or Fisher’s exact test. *SDT* symptom-to-door time, *SD* standardized mean difference, *LVEF* left ventricular ejection fraction, *BMI* body mass index, *SBP* systolic blood pressure, *DBP,* diastolic blood pressure, *PCI* percutaneous coronary intervention, *MI* myocardial infarction, *CABG* coronary artery bypass graft, *HF* heart failure, *CK-MB* creatine kinase myocardial band, *Hs-CRP* high sensitivity C-reactive protein, *HDL* high-density lipoprotein, *LDL* low-density lipoprotein, *GRACE* Global Registry of Acute Coronary Events, *BBs* ß-blockers, *ACEIs* angiotensin-converting enzyme inhibitors, *ARBs* angiotensin receptor blockers, LAD left anterior descending artery, *LCx* left circumflex artery, *RCA* right coronary artery, *ACC/AHA* American College of Cardiology/American Heart Association, *TIMI* thrombolysis in myocardial infarction, *GP* glycoprotein, *IVUS* intravascular ultrasound, *OCT* optical coherence tomography, *FFR* fractional flow reserve, *ZES* zotarolimus-eluting stent, *EES* everolimus-eluting stent, *BES* biolimus-eluting stent. ^*^Drug-eluting stents were composed of ZES (Resolute integrity stent; Medtronic, Inc., Minneapolis, MN), EES (Xience Prime stent, Abbott Vascular, Santa Clara, CA; or Promus Element stent, Boston Scientific, Natick, MA), and BES (BioMatrix Flex stent, Biosensors International, Morges, Switzerland; or Nobori stent, Terumo Corporation, Tokyo, Japan).

|  | Variance Inflation Factors | Tolerance | Condition Index |
| --- | --- | --- | --- |
| Male | 1.363 | 0.734 | 1.000 |
| Age | 3.450 | 0.290 | 3.148 |
| LVEF | 1.290 | 0.775 | 3.553 |
| SBP | 2.935 | 0.341 | 3.788 |
| DBP | 2.581 | 0.387 | 3.932 |
| DBT | 1.176 | 0.850 | 3.991 |
| Atypical chest pain | 1.188 | 0.842 | 4.202 |
| Dyspnea | 1.172 | 0.853 | 4.258 |
| Q-wave in EKG | 1.071 | 0.934 | 4.320 |
| ST-segment depression | 1.961 | 0.510 | 4.390 |
| T-wave inversion | 1.188 | 0.842 | 4.589 |
| Killip class II/III | 1.699 | 0.588 | 4.718 |
| EMS | 1.165 | 0.859 | 4.900 |
| Non-PCI center | 1.189 | 0.841 | 5.111 |
| Hypertension | 1.160 | 0.862 | 5.246 |
| Diabetes mellitus | 1.407 | 0.711 | 5.311 |
| Previous stroke | 1.043 | 0.959 | 5.432 |
| Current smoker | 1.380 | 0.725 | 5.754 |
| Peak CK-MB | 1.143 | 0.875 | 5.913 |
| Peak troponin-I | 1.197 | 0.835 | 6.167 |
| Blood glucose | 1.352 | 0.739 | 7.315 |
| Total cholesterol | 7.038 | 0.142 | 9.031 |
| Triglyceride | 1.504 | 0.665 | 11.157 |
| HDL-cholesterol | 1.429 | 0.700 | 12.942 |
| LDL-cholesterol | 6.062 | 0.165 | 15.631 |
| GRACE risk score | 5.709 | 0.175 | 17.885 |
| Ticagrelor | 1.062 | 0.942 | 23.478 |
| Angicoagulant | 1.049 | 0.953 | 43.406 |
| LCx (IRA) | 1.097 | 0.912 | 51.432 |
| Transradial approach | 1.119 | 0.894 | 57.537 |
| BES | 1.046 | 0.956 | 62.999 |

**Table S3.** Results of the collinearity test for MACE between the SDT <24 h and SDT ≥24 h groups. *MACE* major adverse cardiac events, *LVEF* left ventricular ejection fraction, *SBP* systolic blood pressure, *DBP* diastolic blood pressure, *DBT* door-to-balloon time, *EKG* electrocardiogram, *EMS* emergency medical service, *PCI* percutaneous coronary intervention, *CK-MB* creatine kinase myocardial band, *HDL* high-density lipoprotein, *LDL* low-density lipoprotein, *GRACE* Global Registry of Acute Coronary Events, *LCx* left circumflex coronary artery, *IRA* infarct-related artery, *BES* biolimus-eluting stent.

|  | Variance Inflation Factors | Tolerance | Condition Index |
| --- | --- | --- | --- |
| Male | 1.344 | 0.745 | 1.000 |
| Age | 2.768 | 0.361 | 3.329 |
| LVEF | 1.232 | 0.815 | 3.458 |
| DBP | 1.235 | 0.810 | 3.733 |
| DBT | 1.139 | 0.880 | 4.071 |
| Atypical chest pain | 1.168 | 0.857 | 4.517 |
| Dyspnea | 1.219 | 0.820 | 4.620 |
| ST-segment depression | 1.545 | 0.647 | 4.635 |
| Killip class II/III | 1.655 | 0.605 | 4.814 |
| EMS | 1.031 | 0.970 | 4.917 |
| Hypertension | 1.170 | 0.855 | 5.009 |
| Diabetes mellitus | 1.424 | 0.702 | 5.394 |
| Current smoker | 1.365 | 0.734 | 5.487 |
| Peak CK-MB | 1.177 | 0.850 | 5.578 |
| Peak troponin-I | 1.200 | 0.833 | 6.002 |
| Blood glucose | 1.368 | 0.731 | 6.053 |
| Total cholesterol | 1.322 | 0.751 | 6.160 |
| Triglyceride | 1.235 | 0.810 | 6.760 |
| HDL-cholesterol | 1.233 | 0.812 | 6.934 |
| GRACE risk score | 4.138 | 0.242 | 7.261 |
| LAD (IRA) | 10.212 | 0.098 | 8.635 |
| LCx (IRA) | 8.897 | 0.112 | 9.309 |
| RCA (IRA) | 10.218 | 0.096 | 9.382 |
| LAD (treated vessel) | 2.276 | 0.439 | 9.820 |
| LCx (treated vessel) | 2.191 | 0.456 | 11.510 |
| RCA (treated vessel) | 3.025 | 0.331 | 11.640 |
| ACC/AHA type B2/C lesions | 1.119 | 0.894 | 12.454 |
| GP IIb/IIIa inhibitor | 1.052 | 0.950 | 12.796 |
| Transradial approach | 1.073 | 0.932 | 17.222 |
| IVUS/OCT | 1.099 | 0.910 | 20.153 |
| FFR | 1.015 | 0.986 | 23.525 |
| EES | 1.468 | 0.681 | 24.470 |
| BES | 1.479 | 0.676 | 26.917 |
| Stent diameter | 1.191 | 0.839 | 32.470 |
| Stent length | 2.772 | 0.361 | 48.525 |
| Number of stent | 2.494 | 0.401 | 86.773 |

**Table S4.** Results of the collinearity test for MACE between the complex and non-complex groups *MACE* major adverse cardiac events, *LVEF* left ventricular ejection fraction, *DBP* diastolic blood pressure, *DBT* door-to-balloon time, *EMS* emergency medical service, *CK-MB* creatine kinase myocardial band, *HDL* high-density lipoprotein, *GRACE* Global Registry of Acute Coronary Events, *LAD* left anterior descending coronary artery.
